# Artificial intelligence predicts the immunogenic landscape of SARS-CoV-2 leading to universal blueprints for vaccine designs

**DOI:** 10.1038/s41598-020-78758-5

**Published:** 2020-12-23

**Authors:** Brandon Malone, Boris Simovski, Clément Moliné, Jun Cheng, Marius Gheorghe, Hugues Fontenelle, Ioannis Vardaxis, Simen Tennøe, Jenny-Ann Malmberg, Richard Stratford, Trevor Clancy

**Affiliations:** 1 NEC OncoImmunity AS, Ullernchausseen 64/66, 0379 Oslo, Norway; 2 NEC Laboratories Europe GmbH, Kurfuersten-Anlage 36, 69115 Heidelberg, Germany

**Keywords:** Applied immunology, Vaccines, Data integration, Virtual drug screening, MHC class I, MHC class II, Antigen processing and presentation, Immunotherapy, Immunization, Biotechnology, Computational biology and bioinformatics, Immunology

## Abstract

The global population is at present suffering from a pandemic of Coronavirus disease 2019 (COVID-19), caused by the novel coronavirus Severe Acute Respiratory Syndrome Coronavirus 2 (SARS-CoV-2). The goal of this study was to use artificial intelligence (AI) to predict blueprints for designing universal vaccines against SARS-CoV-2, that contain a sufficiently broad repertoire of T-cell epitopes capable of providing coverage and protection across the global population. To help achieve these aims, we profiled the entire SARS-CoV-2 proteome across the most frequent 100 HLA-A, HLA-B and HLA-DR alleles in the human population, using host-infected cell surface antigen presentation and immunogenicity predictors from the* NEC Immune Profiler *suite of tools, and generated comprehensive epitope maps. We then used these epitope maps as input for a Monte Carlo simulation designed to identify statistically significant “epitope hotspot” regions in the virus that are most likely to be immunogenic across a broad spectrum of HLA types. We then removed epitope hotspots that shared significant homology with proteins in the human proteome to reduce the chance of inducing off-target autoimmune responses. We also analyzed the antigen presentation and immunogenic landscape of all the nonsynonymous mutations across 3,400 different sequences of the virus, to identify a trend whereby SARS-COV-2 mutations are predicted to have reduced potential to be presented by host-infected cells, and consequently detected by the host immune system. A sequence conservation analysis then removed epitope hotspots that occurred in less-conserved regions of the viral proteome. Finally, we used a database of the HLA haplotypes of approximately 22,000 individuals to develop a “digital twin” type simulation to model how effective different combinations of hotspots would work in a diverse human population; the approach identified an optimal constellation of epitope hotspots that could provide maximum coverage in the global population. By combining the antigen presentation to the infected-host cell surface and immunogenicity predictions of the *NEC Immune Profiler* with a robust Monte Carlo and digital twin simulation, we have profiled the entire SARS-CoV-2 proteome and identified a subset of epitope hotspots that could be harnessed in a vaccine formulation to provide a broad coverage across the global population.

## Introduction

The outbreak of Coronavirus disease 2019 (COVID-19) and its rapid worldwide transmission resulted in the World Health Organization (WHO) declaring COVID-19 as a pandemic and global health emergency^1^. COVID-19 is caused by the novel coronavirus Severe Acute Respiratory Syndrome Coronavirus 2 (SARS-CoV-2)^2^. Like all *Coronaviridae*, SARS-CoV-2 is a positive-sense RNA virus encapsulated by an envelope, and characterized by an exposed spike glycoprotein (S-protein) that is projected from the viral surface^3^*.* Although the main structural proteins on *Coronaviridae*, such as the S-protein, are reasonably well studied, many of the other proteins are less well characterized. Correcting this gap may be important to improve the design of therapeutic interventions^4^. This particular gap in knowledge is very relevant from the perspective of finding immunogenic targets across the entire virus proteome, in order to guide the design of effective vaccines. The SARS-CoV-2 virus is closely related in sequence identity and receptor binding to SARS-CoV^5,6^, and therefore it has been purported that one may borrow from this similarity to validate targets in potential vaccines^7,8^. Much of the emphasis on *Coronaviridae* vaccines to date has focused on antibody responses against the S-protein, which is the most “antibody exposed” structural protein in the virus. Although demonstrated to be effective with short-lived responses in a mouse study^9^, the immune response against the S-protein of SARS-CoV is associated with low neutralizing antibody titers and short-lived memory B cell responses in recovered patients^10,11^. Additionally, potential harmful effects of vaccines based on the antibody response to S-protein in SARS-CoV have raised possible safety concerns regarding this approach. For example, in a macaque model, it was observed that anti-S-protein antibodies caused severe acute lung injury^12^, and sera from SARS-CoV patients also revealed that elevated anti-S-protein antibodies were observed in those patients that succumbed to the infection^12^. When considering antibody responses to the S protein, it is also important to consider the possibility that antibody-dependent enhancement (ADE) may occur, whereby antibodies facilitate viral entry into host cells and enhance the infection and inflammation pathology of the virus^13–15^. Considering the potential for ADE, the reported short-lived protective antibody response reported for SARS-CoV^10,11,16^, and that of the seasonal *Coronavridae*^17,18^ and SARS-CoV-2^17,19,20^ , coupled with the pathological consequence of S-protein specific antibodies in certain animal models; it is worth considering diverse strategies for vaccine development that also drive T-cell responses from targets other than the S-protein when designing *Coronaviridae* vaccines^17,21,22^.

In senior citizens, T cell mediated immunity has been shown to a more reliable correlate of protective vaccination^23^. In cases of weakened or a waning antibody response it has been clearly demonstrated that a vaccine strategy that induces both neutralizing antibodies and T cell mediated immunity provides the optimal protection^24^. Additionally, it is possible, due to the waning immune response generated by “antibody alone” driven vaccines, that the first round of immunized populations may also benefit from 2nd generation vaccines that may also explicitly incorporate a broad T cell response^21^.

Although T cells cannot prevent the initial entry of a virus into host cells, they can provide protection by recognizing viral peptides presented by human leukocyte antigens (HLAs) on the surface of host-infected cells or antigen presenting cells (APCs). Several studies have demonstrated in SARS-CoV that virus-specific CD8 T cells are required for mounting an effective immune response and viral clearance^10,25–29^. A vaccine design that confers optimal protection may also need to involve the generation of memory T cell responses^30^. It has been shown that the activation of memory T cells specific for a conserved epitope shared by SARS-CoV and MERS-CoV is a potential strategy for developing coronavirus vaccines^31^. In addition, levels of memory T cell responses to SARS-CoV against peptides from its structural proteins were detected in a proportion of SARS-recovered patients, years after infection^26,32–34^, and most recently detected up to as much as 17 years post infection in 100% of tested patients^35^. Studies of the cross-reactive T cell epitopes in the common cold *Coronaviridae* speculated durable and protective T cell memory responses against SARS-CoV-2^36,37^. For SARS-CoV-2 specific responses, a study of the T cell kinetics revealed that virus specific T cells are present early and increase over time^38^, and these SARS-CoV-2 specific T cell responses are overwhelmingly beneficial Th1 based responses^39^. SARS-CoV-2 specific T cells are clearly important in the elimination of the virus and controlling COVID-19 progression, which lends support to including T cell responses in the design of COVID-19 vaccines^17,22,39^.

However, a T cell response, in isolation, may not be sufficient to combat SARS-CoV-2 infections^22^. The importance of an accompanied neutralizing antibody response with SARS-CoV-2 specific T cell responses has already been demonstrated in cohorts of convalescent patients^40^. In a study of 128 recovered SARS-CoV patients, the immune correlates of protection were investigated and broad CD8, CD4 and neutralizing antibody response were all shown to contribute to protection^41^. The CD4 T cell responses mainly clustered in the S-protein, presumably as B cell antibody responses to the S-protein require the help of CD4 T cells specific to the same protein^42^. Given that in the before-mentioned study^42^ , neutralizing antibody responses correlated with CD8 T cell responses against a broad set of CD8 T cell epitopes in the S-protein, a vaccine design that centers on the S-protein or any other viral protein will need to stimulate a broad CD8 response^43^. In the previous study^43^, robust T cell responses correlated significantly with higher neutralizing antibody activity, consistent with the hypothesis that T cells play an important role in the generation of antibody responses in recovered SARS-CoV patients^41^.

The required CD4 T cell help for a both protective antibody response and protective CD8 T cell activation has been previously well described^44^. The importance of an integrative antibody, CD8 and CD4 T cell response in mounting a successful immune response against the present SARS-CoV-2 threat was well established in a case study during an early stage of the COVID-19 pandemic^45^. This important correlation of protective antibody and T cell response was later confirmed in larger cohorts of convalescent COVID-19 patients^40,46^. Indeed, it is now well established that activated CD4 T cells and CD8 T cells in concert with antibodies against SARS-CoV-2 are recruited in successful protective immune responses against the SARS-CoV-2 virus^41,45,46^.

Many of the previous SARS-CoV studies have found promising CD8 targets^10,25,28,41^, including sustainable memory T cell responses^10,25–27,30–33^ that recognize epitopes in proteins across the entire spectrum of the virus, although the S-protein has been reported to be enriched for dominant CD8 T cell responses^41^. Vaccines that incorporate full length proteins, or attenuated viruses may allow for the patient’s own T cell immunity to sample a broad spectrum of epitopes in a natural manner. However, with whole protein or attenuated viruses, one loses the specificity offered by a targeted T cell epitope-based approach. By predicting virus specific epitopes in a vaccine, highly specific T cell responses can be induced. The computational prediction of specific T cell epitopes minimizes the risk of antigenic competition, the unwanted inclusion of inhibitory epitopes, and is generally considered safer^47^.

Taken together, this supports the approach taken in this study, which is to computationally map a broad epitope landscape across the global viral SARS-CoV-2 proteome, which includes integrated CD8 and CD4 T cell targets in the modeling. There has been some preliminary efforts recently that describe SARS-CoV-2 epitope maps^48–51^, however it appears that the emphasis in those approaches were based mostly on HLA binding. It is important to profile, as in this study, not only the candidates that may bind to HLA but also those CD8 epitopes that are naturally processed and presented by the cell’s antigen processing (AP) machinery and presented on the surface of the infected host cells. Layered on top of the antigen presentation predictions in the host infected cells, we also make predictions across the entire viral proteome that measure the likelihood that the peptides presented on the host infected cells are capable of being recognized by T cells that are not yet tolerized or deleted from a patient’s T cell repertoire. Cross reactivity to the seasonal *Coronaviridae* should also be taken into consideration in this regard in future studies, when considering the pre-existing patient’s T cell repertoire^36,52^. The subsequent immunogenic landscape of the SARS-CoV-2 that we present here is taken further to analyze the immunogenicity of all the non-synonymous variations across approximately 3400 different SARS-CoV-2 sequences, to map the trajectory of differential immunogenic potential between all the currently sequenced viral strains.

Any viable vaccine to tackle SARS-CoV-2 that incorporates T cell epitopes in its design would need to contain a constellation of overlapping epitopes that protect the vast majority of the human HLA population against the virus. In this study, we demonstrate that the SARS-CoV-2 immunogenic landscape clusters into distinct groups across the spectrum of HLA alleles in the human population. Our predicted immunogenic landscape of the SARS-CoV-2 virus is then processed through a robust comprehensive statistical Monte Carlo simulation, incorporating the integrative immune parameters, to identify epitope hotspots for a broad adaptive immune response across the most common HLA genetic makeup in the human population. The central question that the Monte Carlo simulation attempted to answer is whether specific regions in the viral proteins are enriched with higher immunogenic scores with respect to a large set of HLA alleles in the human population, more than expected by chance. In addition, epitope hotspots containing viral epitopes that have high similarity with human peptides, especially those expressed in critical organs were removed. The resulting epitope hotspots we identified represent areas in the viral proteome that are likely to be viable vaccine targets and represent blueprints for vaccine design.

In order to rank-prioritize these potential universal epitope hotspots, and the peptides that underlie them at high resolution, the baseline peptide predictions are then taken through a graph-based “digital twin” type simulation^53^, to prioritize hotspots and the specific overlapping peptides that they comprise at a patient-specific and population-specific level. In this context, the digital twin information is the precise HLA haplotype of an individual, and many virtual individuals are considered within a given population being analyzed. The HLA haplotype is a key determinant of the immune response that specific individuals can mount against SARS-CoV-2 infections^51,54,55^. This HLA genetic background is an important factor for determining whether a vaccine is effective in establishing immunity for the specific individual and a broader population (consisting of multiple diverse individuals). The candidate sequence targets that emerge from this computational analysis represent blueprints for potential vaccine designs modeled across the global human population.

## Results

### The immunogenic landscape of SARS-CoV-2 reveals diversity among the different HLA groups in the human population

**Figure 1:**
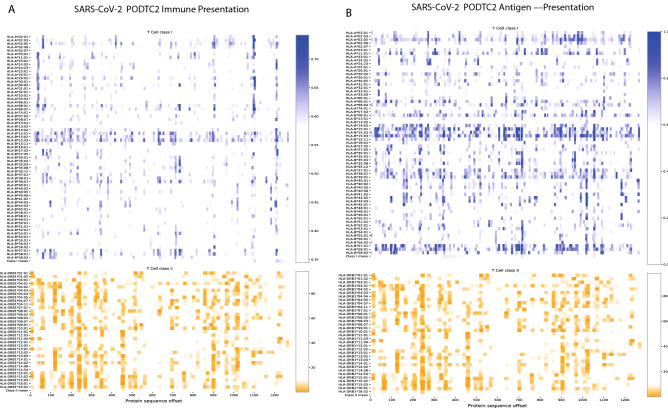
Epitope map of the S-Protein (PODTC2) across the most frequent HLA-A, HLA-B and HLA-DRB alleles in the human population. Data is transformed such that a positive results for CD8 relates to 0.7 or above, and 0.1 or below for Class II. Broad coverage for CD8 and CD4 is demonstrated. PODTC2 is the Uniprot accession ID for the S protien (spike glycoprotien of SARS-CoV-2).

**Figure 2:**
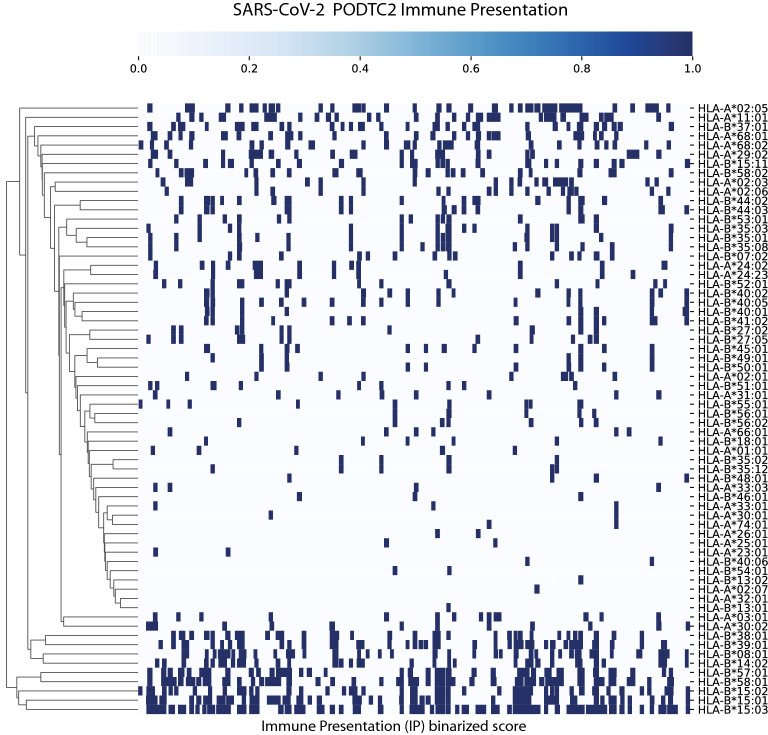
Hierarchical clustering of binary transformation of the epitope maps. Illustrated here for Class I CD8 epitopes in HLA-A and HLA-B alleles. Predictions for Class I greater than 0.7 are transformed to 1. Illustrated for the S-Protein here for demonstration purposes.

We carried out an epitope mapping of the entire SARS-CoV-2 virus proteome using cell-surface antigen presentation and immunogenicity predictors from the *NEC Immune Profiler* suite of tools. Antigen presentation (AP) was predicted from an ensemble machine-learning model that integrates information from several HLA binding predictors (in this case three distinct HLA binding predictors trained on IC50nm binding affinity data) and 13 different predictors of antigen processing (all trained on mass spectrometry data, see section “Generation of global epitope maps and amino acid scores ” in Materials and Methods). The outputted AP score ranges from 0 to 1, and that was used as input to compute immune presentation (IP) across the epitope map. The IP score penalizes those presented peptides that have degrees of “similarity to human” when compared against the human proteome, and awards peptides that are less similar. The resulting IP score represents those HLA-presented peptides that are likely to be recognized by circulating T cells in the periphery, that is, T cells that have not been deleted or tolerized, and therefore most likely to be immunogenic. Both the AP and the IP epitope prediction models are “pan” HLA, or HLA-agnostic, and can be carried out for any allele in the human population; however for the purpose of this study we limited the analysis to the 100 most frequent HLA-A, HLA-B and HLA-DR alleles in the human population (as documented in the Allele Frequency Net Database^56^). Class II HLA binding predictions were also incorporated into the large scale epitope screen from the IEDB consensus of tools^57^. The resulting epitope maps allowed for the identification of regions in the viral proteome that are most likely to be presented by host-infected cells using the most frequent HLA-A, HLA-B and HLA-DR alleles in the global human population (defined conceptually for the purpose of this study as the 100 most frequent HLA-A, HLA-B and HLA-DR alleles in the human population documented in the Allele Frequency Net Database^56^). Epitope maps were created for all of the viral proteins, and an example based on the IP scores for the S-protein is depicted in Fig. 1A and for AP in Fig. 1B; it illustrates distinct regions of the S-protein that contain candidate CD8 and CD4 epitopes for the 100 most frequent human HLA-A, HLA-B and HLA-DR alleles. It is clear from Fig. 1A,B that different HLA alleles have different Class I AP (and IP) and Class II binding properties. This strongly suggests, as one might anticipate, that the SARS-CoV-2 antigen presentation (and immune presentation) landscape clusters into distinct population groups across the spectrum of different human HLA alleles. This trend is further illustrated in the hierarchical-clustering map presented in Fig. 2 after the AP scores have been binarized. Figure 2 clearly demonstrates that some allelic clusters present many viral targets to the human immune system, while others only present a few targets, and some are unable to present any. This implies that different groups in the human population with different HLAs will respond differentially to a T cell driven vaccine composed of viral peptides. Therefore, in order to design the optimal vaccine that leverages the benefits of T cell immunity across a broad human population, we need to predict "epitope hotspots" in viral proteome. These hotspots are regions of the virus that are enriched for overlapping epitopes, and/or epitopes in close spatial proximity, that can be recognized by multiple HLA types across the human population.

Prior to applying the *NEC Immune Profiler* suite of tools to map the SARS-CoV-2 viral proteome, it was important to first validate, to the extent that is possible from the limited number of validated SARS-CoV viral epitopes, that the T cell based AP and IP scores are predicting viable targets. We identified Class I epitopes from the original SARS-CoV virus (that first emerged in the Guangdong province in China in 2002) that shared ≥ 90% sequence identity with the current SARS-CoV-2. Unfortunately, many of the published epitopes were identified using ELISPOT on PBMCs from convalescent patients and/or healthy donors (or humanized mouse models) where the restricting HLA was not explicitly deconvoluted. In order to circumvent this problem, we identified a subset of 8 epitopes where the minimal epitopes and HLA restriction had been identified using tetramers^7,36^. In this survey, 7 out of the 8 epitopes tested were identified as positive, i.e., had an IP score of above 0.5 (see Table 1), demonstrating an accuracy of 87%. The IP score ranges between 0 and 1, where 1 indicates the largest probability of an immune response among the candidates.

**Table 1:** Immune Presentation (IP) scores for validated SARS-CoV peptides with high similarity to SARS-CoV-2.

Peptide	Sequence similarity (%)	Parental protein	IP score	Correct prediction
FIAGLIAIV	100	Spike	0.54	Yes
MEVTPSGTWL	100	Nucleoprotein	0.61	Yes
RLNEVAKNL	100	Spike	0.39	No
TLACFVLAAV	100	Membrane	0.54	Yes
KLPDDFTGCV	90	Spike	0.58	Yes
GMSRIGMEV	100	Nucleoprotein	0.59	Yes
LLLDRLNQL	100	Nucleoprotein	0.57	Yes
VVFLHVTYV	100	Spike precursor	0.53	Yes

Although this was a very small test dataset, this provides a degree of confidence that the *NEC Immune Profiler* prediction pipeline can accurately identify good immunogenic candidates and that the epitope hotspots identified by this analysis and subsequent analyses represent interesting targets for vaccine development.

### A robust statistical analysis identifies epitope hotspots for a broad T cell response

**Figure 3:**
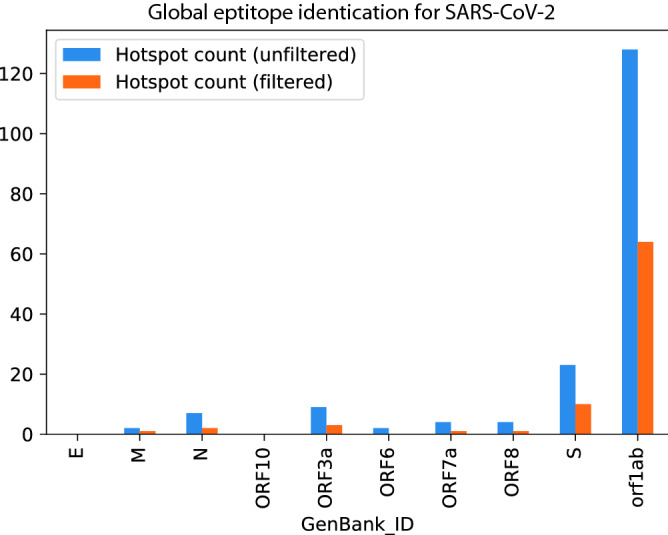
Epitope hotspots from the Monte Carlo analysis are captured across the majority of the entire viral proteome using filtering procedures for conserved and human self-peptides. The most abundant signal for hotspots is in the orf1ab polyprotein.

In order to identify epitope hotspots that have the potential to be viable immunogenic targets for the vast majority of the human population, we first carried out a Monte Carlo random sampling procedure, on the epitope maps generated previously (for the Wuhan reference sequence exemplified in Fig. 1 for the S-protein) to identify specific areas of the SARS-CoV-2 proteome that have the highest probability of being epitope hotspots (see Material and Methods). Three bin sizes were investigated for potential epitope hotspots: 27, 50 and 100. A statistic was calculated for each defined subset region of the protein (bin) from the set of 100 HLAs; the statistic accounts for individual epitope scores and epitope lengths. The Monte Carlo simulation method was then used to estimate the p-values for each bin, whereby each bin represented a candidate epitope hotspot. The epitope hotspots are the statistically significant bins, that is, those below a 5% false discovery rate (FDR) according to the Monte Carlo simulation; these hotspots represent regions that are most likely to contain viable T cell driven vaccine targets that can be recognized by multiple HLA types across the human population. A summary of the epitope hotspots identified across the entire viral proteome for AP is depicted in Fig. 3. It reveals that the most immunogenic regions of the virus, that target the most frequent HLA alleles in the global population, are found in several of the viral proteins above and beyond the antibody exposed structural proteins, such as the S-protein.

### Conservation analysis identifies robust epitope hotspots in SARS-CoV-2

**Figure 4:**
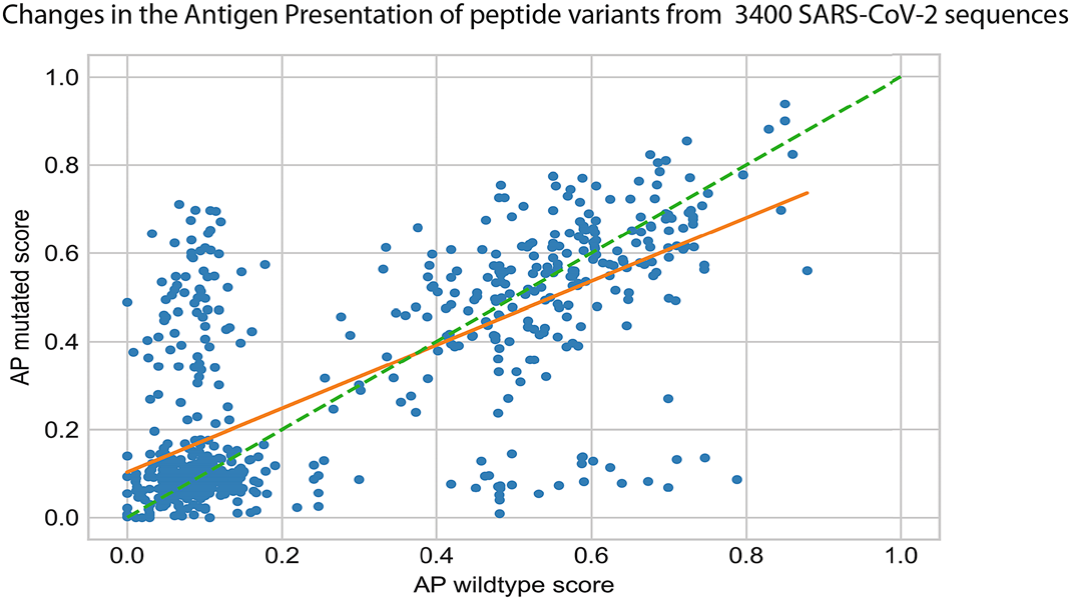
The scatter plot shows the mutated AP score (Y-axis) against its wildtype AP score (X-axis) for each peptide variant. Each data point (blue circles) represents a peptide variant identified in a SARS-COV-2 sequence when comared to the Genbank (Wuhan) reference sequence. There was a total of 1122 peptide variants identified in such a comparison and illustrated on the plot. The orange line is a least square fit with slope = 0.72. Peptide variants on the green dashed line of slope 1 would show no change in AP after introduction of the peptide variant.

Although an reports in demonstrated in a few sequences that the SARS-CoV-2 genome has a lower mutation rate and genetic diversity compared to that of SARS-CoV^58^, another study has demonstrated that there are evolving genetic patterns emerging in different strains of SARS-CoV-2 in diverse geographic locations^59^. A universal vaccine blueprint should ideally protect all populations against different emerging clades of the SARS-CoV-2 virus. Motivated by the necessity to design universal vaccine blueprints that are robust to the mutating nature of SARS-CoV-2, we compared the AP potential of approximately 3,400 virus sequences in the GISAID database against the AP potential of the Wuhan Genbank reference sequence (see Materials and Methods). The outcome of that comparison is illustrated in Fig. 4, and although only illustrated for one HLA allele, hints at a trend whereby SARS-COV-2 peptide variants seem to reduce their potential to be presented and, consequently, detected by the host immune system. Similar trends have been observed in chronic infections such as HPV^60^ and HIV^61^.

On the other hand, a few mutations significantly increase the presentation potential. However, a robust vaccine should ideally target a robust set of hotspots which consistently appear in populations from all geographical regions.

**Figure 5:**
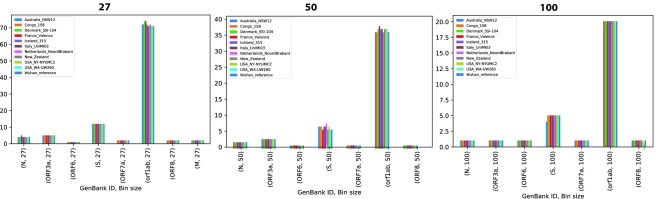
Application of the Antigen presentation (AP) based Monte Carlo epitope hotspot prediction method to mutated viral sequences from 10 different geographical locations, as well as the Wuhan reference sequence. Each group of bars shows the count of epitope hotspots found in the re- spective viral protein in each location. The epitope hotspot counts are shown for three different bin lengths: 27 (left), 50 (centre) and 100 (right). It is clear that the epitope hotspots are robust across mutation sequences. Only those hotposts that were identified for AP in Fig. 3 are illustrated in the plot.

In order to assess if these epitope hotspots are sufficiently robust across sequenced and mutating strains of SARS-CoV-2**,** we next used the AP based epitope hotspot Monte Carlo statistical framework, and analyzed 10 sequences of the virus from among the 10 most mutated viral sequences from different geographical regions^62^. The vast majority of the hotspots were present in all of the sequenced viruses, however occasionally hotspots were eliminated and/or new hotspots emerged in these divergent strains as shown in Fig. 5.

**Figure 6:**
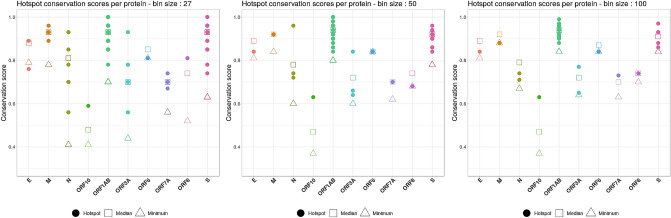
The scatter plots show the distribution of the hotspot conservation scores (Y-axis) for proteins in the viral genome (X-axis). Each plot con- tains the conservation scores of the hotspots identified based on immune presentation (IP), using different bin sizes: 27 (left), 50 (center), and 100 (right). A hotspot is represented by a filled coloured circle, while the median conservation score for a given protein is depicted by a hollow square. As a reference, upwards facing triangles show the minimum conservation score for that protein. Only hotspots with a conservation score above the median were taken for further considertion.

Although the identified hotspots seem to be maintained across different viral strains, in order to design the most robust vaccine blueprint that will provide the broadest protection possible against new emerging clades of the SARS-CoV-2 virus, the epitope hotspots were subject to a sequence conservation analysis. The goal of this analysis was to identify hotspots that appear to be less prone to mutation across thousands of viral sequences, for inclusion into the optimal universal HLA vaccine blueprints, applied to the most conserved regions of the virus. We calculated a conservation score for each hotspot based on the consensus sequence of a protein (see Materials and Methods). Figure 6 shows conservation scores for the hotspots identified based on IP using different bin sizes. Only the epitope hotspots with a conservation score higher than the median conservation score were kept for further analysis. This allowed us to filter out a significant amount of less conserved epitope hotspots, that although have high immunogenic scores, harbor a higher degree of potential sequence variation.

In addition, to reduce the potential for off-target autoimmune responses against host tissue, we removed hotspots that contained exact sequence matches for all epitope lengths analyzed to proteins in the human proteome**.**

### A graph based "digital twin" optimization prioritizes epitopes hotspots to select universal blueprints for vaccine design

**Figure 7:**
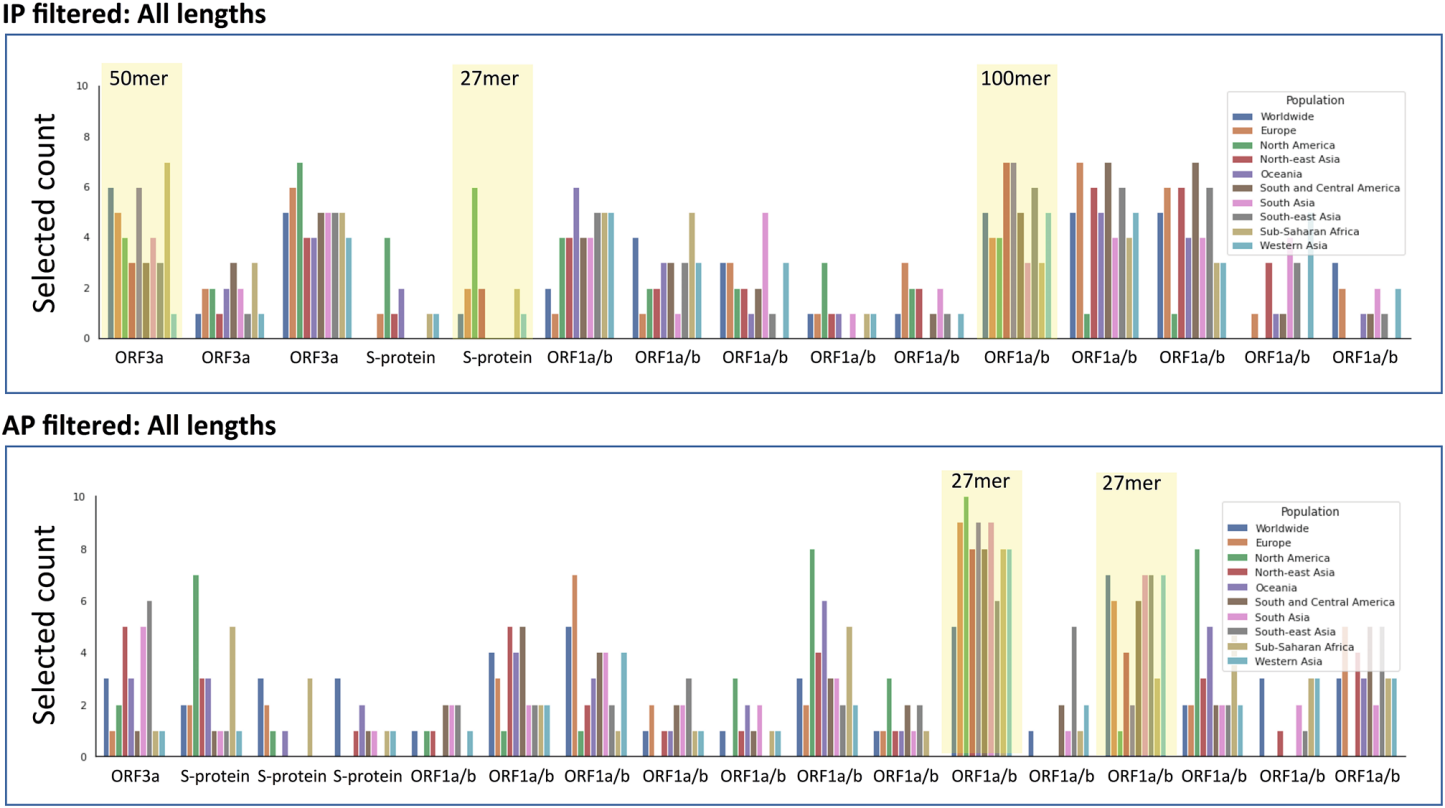
The plot illustrates a set of digital twin simulation experiments to identify effective epitope hotspots based on immune presentation (IP) and antigen presentation (AP), which broadly cover the population. The aim of the analysis was to select an optimum set of hotspots (respecting a given budget) such that the likelihood that each citizen (in a given modelled population) has a positive response is maximized (or, equivalently, that the log likelihood of no response for each citizen is minimized). Ten simulations were run for each region illustrated, where each simulation consisted of 10,000 digital twins (see supplementary file for more details). This plot shows the number of times each hotspot was selected for use in one of the simulations. Each hotspot selected at least 10 times is shown on the x-axis, and the y-axis shows the selected counts per region. Each bar corresponds to a different region-specific simulation setting. A subset of 5 hotspots were selected based on their profile across the AP and IP digital twin analyses, which could theoretically provide coverage of > 90% across a global population (highlighted in yellow).

The Monte Carlo simulation identified well over 100 different hotspots of length 27, 50 or 100 amino acids, for both AP and IP. Even after filtering for conservation and self-similarity, we were left with over 50 different hotpots for both the AP and IP based analyses. In order to develop a blueprint for viable universal vaccine against SARS-CoV-2**,** it is necessary to (1) cover with fidelity a broad proportion of the human population, and (2) prioritize the selection to even fewer regions (the exact number may depend on the size of the bin and the vaccine platform under consideration). Consequently, we need to identify the optimal constellation of hotspots, or relevant viral segments, that can provide broad coverage in the human population with a limited and targeted vaccine “payload”. In order to achieve this aim, we developed and applied (see Materials and Methods) a “digital twin” method, which models the specific HLA haplotypes of different geographical populations. A graph-based mathematical optimization approach is then used to select the optimal combination of immunogenic epitope hotspots that will induce immunity in the broad human population. The results of this analysis are shown in Fig. 7. The output clearly identified a subset of hotspots that may be combined to stimulate a robust immune response in a broad global population. An example hotspot for the ORF3a100-150 region is provided in supplementary Table 1, which shows the amino acid sequence and its component Class I and Class II epitopes.

## Conclusions

In order to effectively combat the SARS-CoV-2 pandemic, a vaccine will need to protect the vast majority of the human population and stimulate diverse T cell responses against multiple viral targets including, but not limited to, the S-protein. To help achieve this ambitious aim, we have profiled the entire SARS-CoV-2 proteome across the most frequent 100 HLA-A, HLA-B and HLA-DR alleles in the human population and generated comprehensive epitope maps. We subsequently used these epitope maps as the basis for modeling the specific HLA haplotype of individual persons in a diverse set of different human populations using the most significant CD8 and CD4 T cell “epitope hotspots” in the virus. To the best of our knowledge, this is the first computational approach that generates comprehensive vaccine design blueprints from large-scale epitope maps of SARS-CoV-2 in a manner that optimizes for diverse T cell immune responses across the global population. Underlying this approach are two novel methods that, when integrated together, result in a solution that is uniquely suited to achieving the objective of the study, i.e., designing blueprints for universal vaccines. Firstly, a framework that leverages Monte Carlo simulations was developed to identify statistically significant epitope hotspot regions in the virus that are most likely to be immunogenic across a broad spectrum of HLA types. Secondly, a novel person-specific or “digital twin” type simulation, based on the actual HLA haplotypes of approximately 22,000 individuals, prioritizes these epitope hotspots, to identify the optimal constellation of vaccine hotspots in the SARS-CoV-2 proteome that are most likely to promote a robust T cell immune response in the global population.

Importantly, the CD8 epitope maps that underlie these optimized epitope hotspots are based on our AP predictions of peptides presented on the surface of host-infected cells, and visible to the host’s CD8 T cells. Additionally, these antigen presented peptides are subject to our IP predictions that infer those specific epitopes that are most likely to activate a T cell in a host’s repertoire that has not been deleted or tolerized. These features confer unique properties to the epitope maps that underlie our epitope hotspot predictions and digital twin optimization. These properties differ from the SARS-CoV-2 epitope maps that have been reported in recent preprints since the outbreak of this virus, which mainly utilize predictions based on HLA binding^48–51^.

A genomic analysis of approximately 3400 SARS-CoV-2 sequences revealed that the epitope hotspots that we predict are robust across different evolving clades of the virus, which may be important in the design of robust vaccine blueprints that are applied to conserved sequences in the virus, and universal to the HLA haplotypes of the global human population. However, on average, mutations in the virus that cause amino acid changes in peptides seem to reduce their potential to be presented on the cell surface and consequently detected by the host immune system. We therefore apply sequence-based filters on the vaccine blueprints that discard less conserved hotspots, and hotspots that harbor peptides that have an exact match in the human proteome, before performing our digital twin simulation.

It is important to note that the predictions derived in this study are in silico based antigen prediction and should be subject to further experimental assay scrutiny^63^, before final definitive selection into a COVID-19 vaccine design. Further research that characterizes potential protective Th1 versus harmful Th2 CD4 T cell responses is critically needed to determine the optimal vaccine design for T cell based vaccines^64^.

The findings described in this study highlight the potential of looking beyond the S-protein and mining the whole viral proteome in order to identify optimal constellations of epitopes that can be used to develop efficacious and universal T-cell vaccines. The novel integrated methodological approaches described in this study may result in the design of diverse T cell driven vaccines that may help combat the SARS-CoV-2 pandemic and bring much needed relief to the suffering global human population**.**

## Materials and methods

### Generation of global epitope maps and amino acid scores

For a given HLA allele, the score allocated to an amino acid corresponds to the best score obtained by an epitope prediction overlapping with this amino acid. For Class I HLA alleles, the epitope lengths are 8, 9, 10 and 11, and predicted for antigen presentation (AP) or immune presentation (IP) of the viral peptide to host-infected cell surface, generated using the NEC Immune Profiler software. These Class I scores range between 0 and 1, where by 1 is the best score, i.e*.*, higher likelihood of being naturally presented on the cell surface (AP) or being recognized by a T cell (IP). For Class II HLA alleles, we only consider 15mers. The Class II predictions were percentile rank binding affinity scores (not antigen presentation), so the lower scores are best (the scores range from 0 to 100, with 0 being the best score).

The NEC Immune Profiler is software based on machine learning algorithms, which predict which antigens have the required features of HLA-binding, processing, presentation to the cell surface, and the potential to be recognized by T cells to be good clinical targets for immunotherapy. The main machine-learning components of the NEC Immune Profiler will be further detailed in the following sections.

- 1. HLA Binding: The ability of an antigen to bind HLA represents the most important step in determining immunogenicity, as only HLA-bound peptides can be detected by circulating T-cells. The NEC Immune Profiler HLA binding module predicts binding affinity of the peptide to the inputted HLA allele(s). The binding affinity predictions are measured by IC50 (nM) scores. The lower the IC50 score, the stronger the peptide binds to the HLA molecule. The module is composed of three different binding affinity predictors.
- 2. Processing: In order to have an opportunity to bind HLA and be subsequently presented at the surface, an antigen must be generated by proteasomal cleavage of its parental polypeptide/protein in the cytosol and be subsequently transported into the endoplasmic reticulum by the TAP transporters. The processing module consists of a series of Support Vector Machines (SVM), trained on large databases of mass spectrometry immunopeptidome data, that are incorporated into the NEC Immune Profiler Software and operate in an ensemble machine learning layer of 13 processing models to predict which antigens have the right physicochemical features to be efficiently processed by the processing apparatus. The different algorithms work in concert to produce a consensus score that ranges between 0 and 1. A consensus score of 1 means that the antigen is predicted to be efficiently processed while conversely a score of 0 means that that the antigen is predicted to be poorly processed.
- 3. Antigen Presentation (AP) and Immune Presentation (IP): In order to stimulate a T-cell a candidate antigen must be presented at the surface of the tumor complexed with HLA. The most important variables that determines whether an antigen will be efficiently presented are: (1) the binding affinity between the candidate antigen and a specific HLA molecule, (2) its potential to be efficiently processed by the antigen processing machinery, (3) the level of expression of the protein containing the mutation and (4) the ability of the source protein to contribute component peptides to the antigen processing pathway. The immune presentation (IP) method generates a distance measure that determines the relative uniqueness of the candidate antigens and can be used in combination with the antigen presentation score to generate an immune presentation (IP) score. Both the AP and IP scores range between 0 and 1. For AP, a score of 1 indicates the largest chance of presentation on the host infected cell surface, and sores > 0.7 are generally considered acceptable. For IP, a score of 1 indicates the largest chance of an immune response among the peptide candidates, and sores > 0.5 are generally considered acceptable. These thresholds were identified as acceptable scores in benchmarking of the NEC Immune Profiler on numerous clinical datasets, whereby the optimal trade-off between specificity and sensitivity in terms of identifying immunogenic epitopes was identified (manuscript in preparation).

### Statistical framework for the detection of epitope hotspot epitope regions in different HLA populations

#### Input data

The data sets inputted into the statistical framework are epitope maps generated for each amino-acid position in all the proteins in the SARS-CoV-2 proteome, for all of the studied 100 HLA alleles (SARS-CoV-2 sequences from the GenBank Wuhan reference, MN908947.3, were downloaded April 15th 2020). A score for any given amino acid was determined as the maximum AP or IP score that a peptide overlapping that amino acid holds in the epitope map. All peptide lengths of size 8–11 amino acids for Class I, and 15 for Class II were processed, generating one HLA dataset per viral protein. Each row in the dataset represents the amino acid epitope scores predicted for one HLA type.

#### Statistical framework

The central question that the statistical framework attempts to answer is: “are specific regions in a given viral protein enriched with higher immunogenic scores, with respect to a given set of HLA types, more than expected by chance?” To answer the question, we implemented a hypothesis-testing framework inspired by work done in statistical genomics^65,66^.

#### HLA tracks

The raw input datasets are first transformed into binary tracks. For each Class I HLA dataset, the epitope scores are transformed to binary (0 and 1) values, such that amino-acid positions with predicted epitope scores larger than 0.7 for AP, or larger than 0.5 for IP, are assigned the value 1 (positively predicted epitope), and the rest are assigned the value 0. Similarly, for Class II HLA datasets, amino-acid positions with predicted epitope scores smaller than 10 are assigned the value 1, otherwise 0. Each binary track can effectively be presented as a list of segments, intervals of consecutive ones, and gaps, intervals of consecutive zeros. For a description of the AP and IP scores please refer to; “Generation of global epitope maps and amino acid scores”, in Material and Methods.

#### Test statistic

For a group of *K* HLA binary tracks, a test statistic *S*_*i*_ is calculated for each bin *b*_*i*_ of given size *m*, dividing the protein in *n* bins (e.g. *m* = 100 amino-acids for the larger proteins). For a single HLA track *k*, a test statistic s_i,k_ is calculated for each bin *b*_*i*_ as follows.

$${s}_{i,k}={\sum }_{J=1}^{m}{b}_{i,j}*{weight}_{k},$$

where ${weight}_{k}$ is by default 1.0.

Then, for each bin *i* = 1...*n*,

$${S}_{i}=\frac{{\sum }_{k=1}^{K}{s}_{i,k}}{K}$$

which is the average number of amino acids predicted to be included in an epitope (epitope enrichment) of the bin *b*_*i*_ normalized by the number of selected HLAs.

#### Null model

As with genomic tracks^66^, analytical approaches to estimate the statistical significance of the observed enrichment with predicted epitopes, of a region, across multiple HLA tracks, are intractable. An effective alternative to this problem is Monte Carlo-based simulation. A null model is defined as the generative model of the HLA tracks, if they were generated by chance. From the null model, through sampling, arises the null distribution of the test statistic s_i,k_. Amino acids that are positively predicted as epitopes will clump together in segments with minimal length of eight, which is the shortest peptide length for which epitope scores are predicted, and often form longer segments when the source peptides overlap each other. Similarly, non-epitope amino acids will form gaps, with a minimal possible length of one amino acid. To preserve these features of the observed HLA tracks in the null model, as a sampling strategy we selected to shuffle the order of the segments and the gaps, respectively, within an HLA track.

#### P-value estimation

To sample from the null model, all of the *K* HLA tracks are divided in segments and gaps, which are then shuffled to produce a randomized HLA track. This is repeated 10,000 times, to produce 10,000 samples of s_i,k_ statistic for each bin. For each bin, the p-value is estimated as the proportion of the samples that are equal or larger than the truly observed enrichment. Further, the generated p-values are adjusted for multiple testing with the Benjamini–Yekutieli procedure to control for a false discovery rate (FDR) of 0.05.

### Graph-based optimization in digital twin simulations of the epitope hotspots

We consider a population as a set *C* of “digital twin” citizens *c*, and a vaccine as a set *V* of vaccine elements *v*. We denote the likelihood that all citizens have a positive response to a vaccine as $P\left(R=+|C,V\right)$. Our goal is to design a vaccine, that is, select a set of vaccine elements, to maximize this probability:

$$\underset{V}{\mathrm{max}} P\left(R=+|V,C\right)$$

In this setting, maximizing the probability of positive response is the same as minimizing the probability of no response. Thus, we approach vaccine design by minimizing the probability of no response for the citizen who has the highest probability of no response $P\left(R=-|V,{c}_{j}\right)$:

$$\mathop {{\text{max}}}\limits_{V} P\left( {R = + |V,C} \right): = \mathop {{\text{min}}}\limits_{V} \mathop {{\text{max}}}\limits_{{c_{j} \in C}} \left\{ {P\left( {R = - |V,c_{j} } \right)} \right\}$$

We consider that a vaccine causes a positive response if at least one of its elements causes a positive response. That is, the probability of no response is the joint likelihood that all elements fail. For a particular citizen ${c}_{j}$, this probability is given as follows.

$$P\left(R=-|V,{c}_{j}\right)=\prod_{{v}_{i}\in V}P(R=-|v,{c}_{j}, V)$$

The original optimization problem can then be expressed as:

$$\underset{V}{\mathrm{min }}\underset{{c}_{j}\in C}{\mathrm{max}}\prod_{{v}_{i}\in V}P(R=-|{v}_{i},{c}_{j},V)$$

Since the logarithm function is monotonic, the value of *V* which minimizes the logarithm of the function also minimizes the original function.

$$\underset{V}{\mathrm{min }}\underset{{c}_{j}\in C}{\mathrm{max}}\sum_{{v}_{i}\in V}\mathrm{log}P(R=-|{v}_{i},{c}_{j},V)$$

Further, we consider each citizen as a HLA haplotype, and we assume that each vaccine element v_i_ may result in a response for each HLA allele independently; we refer to the alleles for citizen ${c}_{j}$ as $A({c}_{j})$. Thus, our final objective is as follows.

$$\underset{V}{\mathrm{min }}\underset{c\in C}{\mathrm{max}}\sum_{{v}_{i}\in V}\sum_{{a}_{k}\in A(c)}\mathrm{log}P(R=-|{v}_{i},k,V)$$

**Figure 8:**
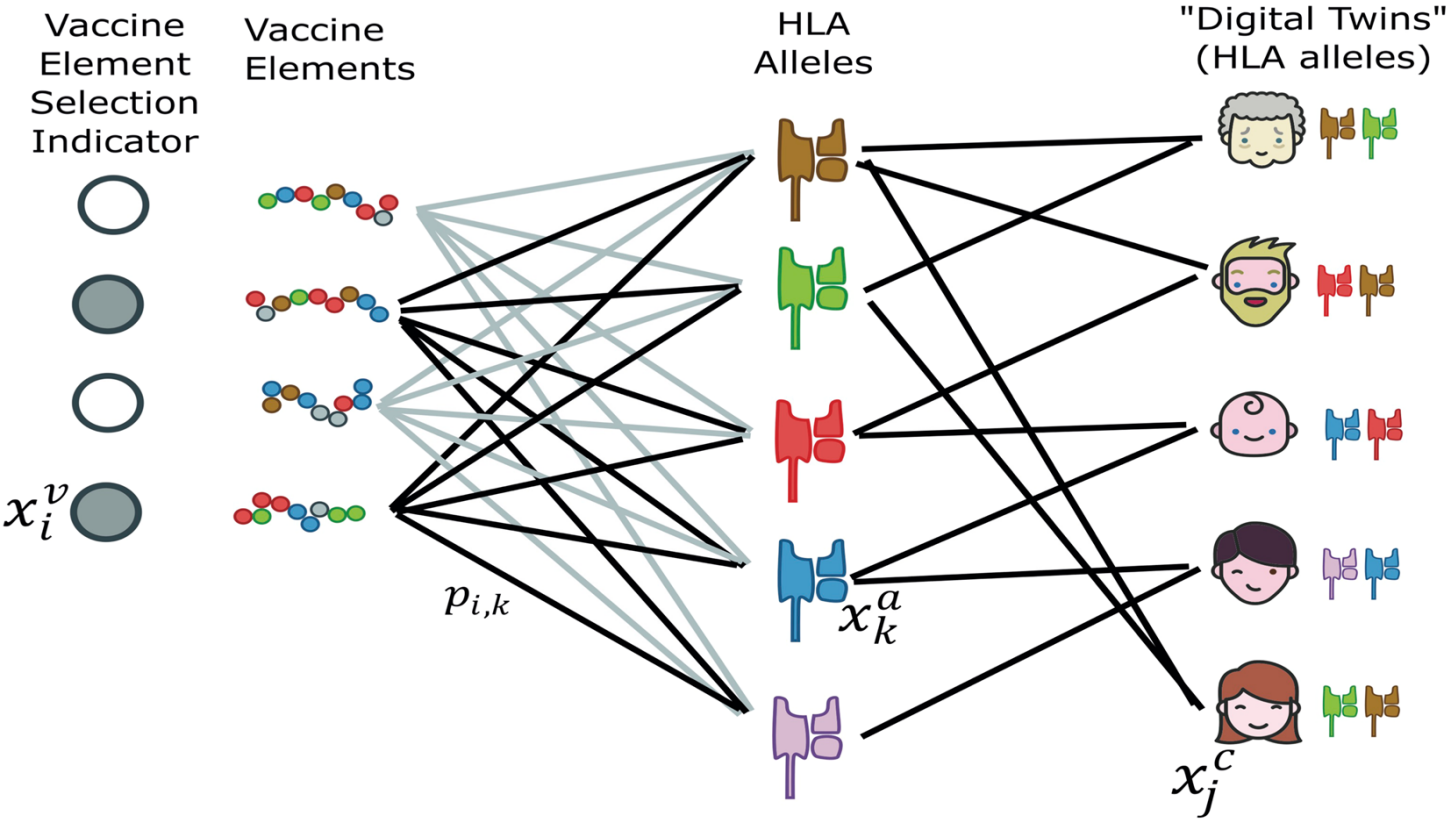
Schematic of the problem setting. The vaccines elements were the significant epitope hotspots that emerged from the statistical hotspot detection framework.

We approach this minimax problem as a type of network flow problem, with one set of nodes corresponding to vaccine elements, one set corresponding to HLA alleles, and one set corresponding to citizens. The goal is to select the set of vaccine elements such that the likelihood of no response is minimized for each citizen. Figure 8 gives an overview of the problem setting. Supplementary table 2 provides an overview of the haplotypes used in the simulations, including the number of unique haplotypes in each geographical region, and supplementary table 3 provides the full details of the complete haplotypes used for each individual in the digital twin simulations.

#### Vaccine design process

Concretely, we approach the vaccine design process in four steps:

- 1. Select a set of candidate vaccine elements for inclusion in the vaccine. The epitope hotspots are the candidate vaccine elements.
- 2. Create a set of “digital twin” citizens for a population of interest, where a digital twin is an HLA haplotype.
- 3. Create a tripartite graph in which the nodes correspond to vaccine elements, HLA alleles, and citizens; edges correspond to relevant biological terms described in the supplementary methods.
- 4. Select a set of vaccine elements (respecting a given budget) such that the likelihood that each citizen has a positive response is maximized (or, equivalently, that the log likelihood of no response for each citizen is minimized).

Each step is described in more detail in the supplementary methods.

### Variant immunogenic potential across the mutating sequences of SARS-CoV-2

We downloaded all the strains available in the GISAID database^62^ as of 31.03.2020, and ran them through the Nexstrain/Augur software suite with default parameters^67^. We parsed the resulting phylogenic tree to obtain all protein variants. For each peptide variant, we computed a wildtype score and a mutated Antigen Presentation (AP) score for HLA-A*02:01. One HLA allele was chosen to illustrate the robustness across mutating sequences of the virus, illustrated in Fig. 4. The mutated score is the maximum AP score among point mutations in the nine possible 9-mer peptides that include the variant. The wildtype score is the maximum AP score for the 9-mers at the same positions in the reference (Wuhan) strain.

### Epitope hotspot conservation scores

For each protein within the viral genome, the set of unique amino acid sequences was compiled from all the strains available in the GISAID database^62^ as of 29.03.2020. These sets were individually processed using the Clustal Omega (v1.2.4)^68^ software via the command line interface with default parameter settings. The software outputs a consensus sequence that contains conservation information for each amino acid within the protein sequence. An amino acid depicted as an “*” at position *i* within the consensus sequence translates to that amino acid being conserved (exact match in sequence similarity) at position *i* among all the input sequences^68^.

The hotspot offsets were then used to extract their respective consensus sub-sequence. For each hotspot, the conservation score was calculated as the ratio of “*”s within its consensus sub-sequence to the total length of the sub-sequence. Accordingly, each hotspot was assigned a conservation score between 0 and 1, with 1 representing a perfect conservation across all available strains.

The median conservation score was calculated by sampling 1,000 sub-sequences equal to the hotspot size from the entire consensus sequence of a protein. Each sample was assigned a conservation score, and the median value from all 1,000 conservation scores was calculated. The minimum conservation score was calculated using a sliding window approach, with the window size being equal to the hotspot size. For each increment, a conservation score was calculated, and the resulting minimum conservation score was kept.

## Supplementary Information

Supplementary Table 1.

Supplementary Table 2.

Supplementary Table 3.

Supplementary Information.

## Data Availability

The data associated with this study is also available at www.Synapse.org (accession: syn22216071), and the method is also downloadable at Protocol Exhange (https://protocolexchange.researchsquare.com/) under the same title and authorship.

## References

[1] WHO. *World Health Organization. WHO Director-General’s opening remarks at the media briefing on COVID-19—11 March* 2020.

[2] Coronaviridae Study Group of the International Committee on Taxonomy of V (2020). The species Severe acute respiratory syndrome-related coronavirus: classifying 2019-nCoV and naming it SARS-CoV-2. Nat. Microbiol..

[3] Barcena M (2009). Cryo-electron tomography of mouse hepatitis virus: Insights into the structure of the coronavirion. Proc. Natl. Acad. Sci. USA.

[4] Fehr AR, Perlman S (2015). Coronaviruses: an overview of their replication and pathogenesis. Methods Mol. Biol..

[5] Zhou P (2020). A pneumonia outbreak associated with a new coronavirus of probable bat origin. Nature.

[6] Lu R (2020). Genomic characterisation and epidemiology of 2019 novel coronavirus: implications for virus origins and receptor binding. Lancet.

[7] Grifoni A (2020). A sequence homology and bioinformatic approach can predict candidate targets for immune responses to SARS-CoV-2. Cell Host Microbe.

[8] Ahmed SF, Quadeer AA, McKay MR (2020). Preliminary identification of potential vaccine targets for the COVID-19 coronavirus (SARS-CoV-2) based on SARS-CoV immunological studies. Viruses.

[9] Yang ZY (2004). A DNA vaccine induces SARS coronavirus neutralization and protective immunity in mice. Nature.

[10] Channappanavar R, Zhao J, Perlman S (2014). T cell-mediated immune response to respiratory coronaviruses. Immunol. Res..

[11] Liu W (2006). Two-year prospective study of the humoral immune response of patients with severe acute respiratory syndrome. J. Infect. Dis..

[12] Liu L (2019). Anti-spike IgG causes severe acute lung injury by skewing macrophage responses during acute SARS-CoV infection. JCI Insight.

[13] Tirado SM, Yoon KJ (2003). Antibody-dependent enhancement of virus infection and disease. Viral Immunol..

[14] Wan Y (2020). Molecular mechanism for antibody-dependent enhancement of coronavirus entry. J. Virol..

[15] Tetro JA (2020). Is COVID-19 receiving ADE from other coronaviruses?. Microbes Infect..

[16] Cao WC, Liu W, Zhang PH, Zhang F, Richardus JH (2007). Disappearance of antibodies to SARS-associated coronavirus after recovery. N. Engl. J. Med..

[17] Sariol A, Perlman S (2020). Lessons for COVID-19 immunity from other coronavirus infections. Immunity.

[18] Edridge AWD (2020). Seasonal coronavirus protective immunity is short-lasting. Nat. Med..

[19] Seow J (2020). Longitudinal observation and decline of neutralizing antibody responses in the three months following SARS-CoV-2 infection in humans. Nat. Microbiol..

[20] Long QX (2020). Clinical and immunological assessment of asymptomatic SARS-CoV-2 infections. Nat. Med..

[21] Jeyanathan M (2020). Immunological considerations for COVID-19 vaccine strategies. Nat. Rev. Immunol..

[22] Tay MZ, Poh CM, Renia L, MacAry PA, Ng LFP (2020). The trinity of COVID-19: immunity, inflammation and intervention. Nat. Rev. Immunol..

[23] Haq K, McElhaney JE (2014). Immunosenescence: influenza vaccination and the elderly. Curr. Opin. Immunol..

[24] Arunachalam PS (2020). T cell-inducing vaccine durably prevents mucosal SHIV infection even with lower neutralizing antibody titers. Nat. Med..

[25] Channappanavar R, Fett C, Zhao J, Meyerholz DK, Perlman S (2014). Virus-specific memory CD8 T cells provide substantial protection from lethal severe acute respiratory syndrome coronavirus infection. J. Virol..

[26] Yang LT (2006). Long-lived effector/central memory T-cell responses to severe acute respiratory syndrome coronavirus (SARS-CoV) S antigen in recovered SARS patients. Clinical Immunol..

[27] Yang L (2007). Persistent memory CD4+ and CD8+ T-cell responses in recovered severe acute respiratory syndrome (SARS) patients to SARS coronavirus M antigen. J. General Virol..

[28] Chen J (2010). Cellular immune responses to severe acute respiratory syndrome coronavirus (SARS-CoV) infection in senescent BALB/c mice: CD4+ T cells are important in control of SARS-CoV infection. J. Virol..

[29] Janice Oh HL, Ken-En Gan S, Bertoletti A, Tan YJ (2012). Understanding the T cell immune response in SARS coronavirus infection. Emerg. Microbes Infect..

[30] Wherry EJ, Ahmed R (2004). Memory CD8 T-cell differentiation during viral infection. J. Virol..

[31] Zhao J (2016). Airway memory CD4(+) T cells mediate protective immunity against emerging respiratory coronaviruses. Immunity.

[32] Fan YY (2009). Characterization of SARS-CoV-specific memory T cells from recovered individuals 4 years after infection. Adv. Virol..

[33] Ng OW (2016). Memory T cell responses targeting the SARS coronavirus persist up to 11 years post-infection. Vaccine.

[34] Libraty DH, O'Neil KM, Baker LM, Acosta LP, Olveda RM (2007). Human CD4(+) memory T-lymphocyte responses to SARS coronavirus infection. Virology.

[35] Le Bert N (2020). SARS-CoV-2-specific T cell immunity in cases of COVID-19 and SARS, and uninfected controls. Nature.

[36] Mateus J (2020). Selective and cross-reactive SARS-CoV-2 T cell epitopes in unexposed humans. Science.

[37] Sette A, Crotty S (2020). Pre-existing immunity to SARS-CoV-2: the knowns and unknowns. Nat. Rev. Immunol..

[38] Weiskopf D (2020). Phenotype and kinetics of SARS-CoV-2-specific T cells in COVID-19 patients with acute respiratory distress syndrome. Sci. Immunol..

[39] Altmann DM, Boyton RJ (2020). SARS-CoV-2 T cell immunity: specificity, function, durability, and role in protection. Sci. Immunol..

[40] Ni L (2020). Detection of SARS-CoV-2-specific humoral and cellular immunity in COVID-19 convalescent individuals. Immunity.

[41] Li CK (2008). T cell responses to whole SARS coronavirus in humans. J. Immunol..

[42] Mitchison NA (2004). T-cell-B-cell cooperation. Nat. Rev. Immunol..

[43] Herst CV (2020). An effective CTL peptide vaccine for Ebola Zaire Based on Survivors' CD8+ targeting of a particular nucleocapsid protein epitope with potential implications for COVID-19 vaccine design. Vaccine.

[44] Chen K, Kolls JK (2013). T cell-mediated host immune defenses in the lung. Annu. Rev. Immunol..

[45] Thevarajan I (2020). Breadth of concomitant immune responses prior to patient recovery: a case report of non-severe COVID-19. Nat. Med..

[46] Grifoni A (2020). Targets of T cell responses to SARS-CoV-2 coronavirus in humans with COVID-19 disease and unexposed individuals. Cell.

[47] Panagioti E, Klenerman P, Lee LN, van der Burg SH, Arens R (2018). Features of effective T cell-inducing vaccines against chronic viral infections. Front. Immunol..

[48] Campbell, K. M., Steiner, G., Wells, D. K., Ribas, A. & Kalbasi, A. Prediction of SARS-CoV-2 epitopes across 9360 HLA class I alleles. *bioRxiv* (2020).

[49] Nguyen, A. *et al.* Human leukocyte antigen susceptibility map for SARS-CoV-2. *medRxiv* (2020). 10.1128/JVI.00510-20 PMC7307149 32303592

[50] Poran, A. *et al.* Sequence-based prediction of vaccine targets for inducing T cell responses to SARS-CoV-2 utilizing the bioinformatics predictor RECON. *bioRxiv* (2020).

[51] Nguyen A (2020). Human leukocyte antigen susceptibility map for severe acute respiratory syndrome coronavirus 2. J. Virol..

[52] Pacheco-Olvera, D. L. *et al.* Bioinformatic analysis of shared B and T cell epitopes amongst relevant coronaviruses to human health: Is there cross-protection? *bioRxiv* (2020).

[53] Bjornsson B (2019). Digital twins to personalize medicine. Genome Med..

[54] Zahn LM (2020). HLA genetics and COVID-19. Science.

[55] Barquera R (2020). Binding affinities of 438 HLA proteins to complete proteomes of seven pandemic viruses and distributions of strongest and weakest HLA peptide binders in populations worldwide. HLA.

[56] Gonzalez-Galarza FF (2020). Allele frequency net database (AFND) 2020 update: gold-standard data classification, open access genotype data and new query tools. Nucleic Acids Res..

[57] Dhanda SK (2019). IEDB-AR: immune epitope database-analysis resource in 2019. Nucleic Acids Res..

[58] Jia Y (2020). Analysis of the mutation dynamics of SARS-CoV-2 reveals the spread history and emergence of RBD mutant with lower ACE2 binding affinity. bioRxiv.

[59] Pachetti M (2020). Emerging SARS-CoV-2 mutation hot spots include a novel RNA-dependent-RNA polymerase variant. J. Transl. Med..

[60] Rosenberg W (1999). Mechanisms of immune escape in viral hepatitis. Gut.

[61] Batorsky R, Sergeev RA, Rouzine IM (2014). The route of HIV escape from immune response targeting multiple sites is determined by the cost-benefit tradeoff of escape mutations. PLoS Comput. Biol..

[62] Shu Y, McCauley J (2017). GISAID: global initiative on sharing all influenza data—from vision to reality. Eur. Commun. Disease Bull..

[63] Paul S (2020). Benchmarking predictions of MHC class I restricted T cell epitopes in a comprehensively studied model system. PLoS Comput. Biol..

[64] Lurie N, Saville M, Hatchett R, Halton J (2020). Developing covid-19 vaccines at pandemic speed. N. Engl. J. Med..

[65] Simovski B (2017). GSuite HyperBrowser: integrative analysis of dataset collections across the genome and epigenome. GigaScience.

[66] Sandve GK (2010). The Genomic HyperBrowser: inferential genomics at the sequence level. Genome Biol..

[67] Hadfield J (2018). Nextstrain: real-time tracking of pathogen evolution. Bioinformatics.

[68] Sievers F, Higgins DG (2018). Clustal omega for making accurate alignments of many protein sequences. Protein Sci. Public. Protein Soc..

