# Supplementary material for: Artificial intelligence predicts the immunogenic landscape of SARS-CoV-2 leading to universal blueprints for vaccine designs
Source: Sci Rep. 2020 Dec 23;10:22375. doi: 10.1038/s41598-020-78758-5 (PMC7758335; doi:10.1038/s41598-020-78758-5)
Supplement: Supplementary file 2 — Supplementary Table 2. [file 41598_2020_78758_MOESM2_ESM.pdf]

Artificial intelligence predicts the immunogenic landscape of SARS-CoV-2 leading to universal blueprints for vaccine designs

Brandon Malone*[2], Boris Simovski*[1], Clément Moliné*[1], Jun Cheng[2], Marius Gheorghe[1], Hugues Fontenelle[1], Ioannis Vardaxis[1], Simen Tennøe[1], Jenny-Ann Malmberg[1], Richard Stratford[1], Trevor Clancy¶[1]

[1]NEC OncoImmunity AS, Oslo Cancer Cluster, Ullernchausseen 64/66, 0379 Oslo, Norway
[2]NEC Laboratories Europe GmbH Kurfuersten-Anlage 36, 69115 Heidelberg, Germany
*These authors contributed equally
¶Corresponding author (Email: trevor@oncoimmunity.com)

Supplementary Table 2: The number of total observed individuals (complete haplotypes) and the number of unique haplotypes observed in each region.

Region	Number of individuals	Number of unique haplotypes
Europe	1393	1308
North America	1075	1010
North-East Asia	104	100
Oceania	197	117
South Asia	3035	2946
South and Central America	2904	2791
South-East Asia	13071	9592
Sub-Saharan Africa	401	398
Western Asia	157	157
