# Supplementary material for: Artificial intelligence predicts the immunogenic landscape of SARS-CoV-2 leading to universal blueprints for vaccine designs
Source: Sci Rep. 2020 Dec 23;10:22375. doi: 10.1038/s41598-020-78758-5 (PMC7758335; doi:10.1038/s41598-020-78758-5)
Supplement: Supplementary file 4 — Supplementary Information. [file 41598_2020_78758_MOESM4_ESM.pdf]

Artificial intelligence predicts the immunogenic landscape of SARS-CoV-2 leading to universal blueprints for vaccine designs

Brandon Malone*[2], Boris Simovski*[1], Clément Moliné*[1], Jun Cheng[2], Marius Gheorghe[1], Hugues Fontenelle[1], Ioannis Vardaxis[1], Simen Tennøe[1], Jenny-Ann Malmberg[1], Richard Stratford[1], Trevor Clancy¶[1]

[1]NEC OncoImmunity AS, Oslo Cancer Cluster, Ullernchausseen 64/66, 0379 Oslo, Norway
[2]NEC Laboratories Europe GmbH Kurfuersten-Anlage 36, 69115 Heidelberg, Germany
*These authors contributed equally
¶Corresponding author (Email: trevor@oncoimmunity.com)

Supplementary methods

The digital twin simulation framework

Step 1. Select a set of candidate vaccine elements

Each identified hotspot is a candidate element of a vaccine. Each candidate vaccine element v_i is associated with a cost c_i^v, while a total budget b is available for including elements in the vaccine. The description of the budget and costs depend on the vaccine platform.

Some vaccine platforms are mainly restricted to a fixed number of vaccine elements; in this case, each cost c_i^v will be 1, and the budget will indicate the total number of elements which can be included.

Some other vaccine platforms are restricted to a maximum length of included elements. In this case, each cost c_i^v will be the length of the vaccine element, and the budget will indicate the maximum length of elements which can be included.

Step 2. Create a set of "digital twin" citizens

Our approach is based on simulating a set of "digital twin" citizens. In this work, we focus on vaccine elements whose effects are determined, in part, by the HLAs of each citizen. Thus, each digital twin corresponds to a set of HLA alleles.

It is known[1] that citizens from different regions of the world tend to have different sets of HLA alleles; further, some combinations of HLA alleles are more common than others. We use full HLA genotypes from actual citizens available from high-quality samples in the Allele Frequency Net Database[2] (AFND) to accurately model these relationships.

Creating a distribution over genotypes for each region. In particular, AFND assigns each sample to a region based on where the sample came from (e.g., "Europe" or "Sub-Saharan

---

[1] Cao, K.; JillHollenbach; Shi, X.; Shi, W.; Chopek, M. & Fernández-Viña, M. A. Analysis of the frequencies of HLA-A, B, and C alleles and haplotypes in the five major ethnic groups of the United States reveals high levels of diversity in these loci and contrasting distribution patterns in these populations. Human Immunology, 2001, 62, 1009-1030.
[2] http://www.allelefrequencies.net/

Africa"). In a first step, we create a posterior distribution over genotypes in each region based on the observations and an uninformative (Jeffreys) prior distribution.

Specifically, we collect all genotypes observed at least once across all regions; we assign an index *g* to each genotype, and we call the total number of unique genotypes as *G*. Second, we specify a prior distribution over genotypes. We use a symmetric Dirichlet distribution with concentration parameter of 0.5 because this distribution is uninformative in an information theoretic sense and does not reflect strong prior beliefs that any particular genotypes are more likely to appear in any specific region. For each region, we then calculate a posterior distribution over genotypes as a Dirichlet distribution as follows.

$$\theta_1, \ldots, \theta_G | x_1, \ldots, x_G \sim Dirichlet(\alpha_1 + x_1, \ldots, \alpha_G + x_G)$$

where α_g is the (prior) concentration parameter for the gth genotype (always 0.5 here) and x_g is the number of times the gth genotype was observed in the region.

We can now use this distribution to sample genotypes from a region using a two-step process.

$$\theta_1, \ldots, \theta_G \sim Dirichlet(\alpha_1 + x_1, \ldots, \alpha_G + x_G)$$

$$y_1, \ldots, y_G \sim Multinomial(\theta_1, \ldots, \theta_G; n)$$

where *n* is the desired number of genotypes to sample from the region, and $y_1, \ldots, y_G$ are the counts of each genotype in the sample.

Creating a set of "digital twin" citizens. We create a set of digital twin citizens using a two-step approach. Our method must be given the population size *p*, as well as a distribution over regions. Concretely, the input is a Dirichlet distribution over the regions, as well as *p*. (We note that this Dirichlet is completely independent of those over genotypes discussed in the previous section.) The number of citizens from each region is sampled using the same two-step sampling process described above.

Second, the genotypes for each region are sampled using the posterior distributions over genotypes discussed above.

Step 3. Create a tripartite graph

We next use the vaccine elements and digital twins to construct a tripartite graph that will form the basis of the optimization problem for vaccine design. The graph has three sets of nodes:

1. All candidate vaccine elements identified in Step 1
2. All HLA alleles in all digital twin genotypes
3. All digital twins

The graph also has two sets of weighted edges:

1. An edge from each vaccine element v_i to each HLA allele a_k. The weight of this edge is $\log P(R = -|v_i, a_k)$, that is, the likelihood of no response for the allele from that particular vaccine element. (We describe below an approach for calculating this value for short peptides.)

2. An edge from each allele to each citizen which has that allele in its genotype. The weight of these edges is always 1.

As an intuition, we call the edges from a vaccine element to an allele (and, then, from the allele to each patient with that allele) as "active" when the vaccine element is selected. Then, the log likelihood of response for a citizen is the sum of all active incoming edges. That is, the flow from selected vaccine elements to the citizens gives the likelihood of no response for that citizen.

$$\sum_{v_i \in V} \sum_{a_k \in A(c_j)} \log P(R = -|v_i, a_k)$$

This definition does not include *V* in the conditioning set of the likelihood. Thus, it does not account for interactions among vaccine elements, such as immunodominance.

Calculating the likelihood of no response for a given digital twin and vaccine elements. We now describe example approaches for calculating $\log P(R = -|v_i, a_k)$ for three types of vaccine elements. Our vaccine design approach is applicable for any approach which assigns a value for $\log P(R = -|v_i, a_k)$.

1. *Short peptide sequences.* Most short peptide prediction engines[3] compute some sort of a score that a peptide will result in some immune response (e.g., binding, presentation, cytokine release, etc.), and this score generally takes into account a specific HLA allele. In some cases, this is already a probability, and in others, it can be converted into a probability using a transformation function, such as a logistic function.

   Thus, the prediction engines give $P(R = +|v_i, a_k)$, where v_i is the peptide and a_k is the allele. We then take $\log P(R = -|v_i, a_k) = \log[1 - P(R = +|v_i, a_k)]$.

2. *Long peptide sequences*. Longer peptide sequences may include multiple short peptide sequences with different scores from the prediction engine. An example approach to calculate $\log P(R = -|v_i, a_k)$, where *v* is the long peptide sequence, is to take the minimum (i.e., best) $\log P(R = -|p, a_k)$, where *p* is any short peptide contained in v_i.

---

[3] Jensen, K. K.; Andreatta, M.; Marcatili, P.; Buus, S.; Greenbaum, J. A.; Yan, Z.; Sette, A.; Peters, B. & Nielsen, M. Improved methods for predicting peptide binding affinity to MHC class II molecules. Immunology, 2018, 154, 394-406.

3. *Longer amino acid sequences*. Longer amino acid sequences may contain even more short peptide sequences, and the same approach used for *long peptide sequences* can be used here.

Step 4. Selecting a set of vaccine elements

Finally, we pose the vaccine design problem as a type of network flow problem through the graph defined in Step 3. In particular, the minimization problem can be posed as an integer linear program (ILP); thus, it can be provably, optimally solved using conventional ILP solvers.

Handling the minimax problem. As previously described, our goal is to choose the set of vaccine elements which minimize the log likelihood of no response for each patient.

$$\min_{V} \max_{c \in C} \sum_{v_i \in V} \sum_{a_k \in A(c_j)} \log P(R = -|v_i, a_k, V)$$

We ignore any interactions among vaccine elements, so the minimax problem simplifies as follows.

$$\min_{V} \max_{c \in C} \sum_{v_i \in V} \sum_{a_k \in A(c_j)} \log P(R = -|v_i, a_k)$$

In practice, we can remove *V* from the conditioning set. Thus, the terms inside the summation are exactly those calculated in Step 3 as the weights on the edges in the graph.

Standard ILP solvers cannot directly solve this minimax problem; however, we use the standard approach of a set of surrogate variables to address this problem. In particular, we define x_j^c to be the log likelihood of no response for citizen c_j. That is, $x_j^c \coloneqq \sum_{v_i \in V} \sum_{a_k \in A(c_j)} \log P(R = -|v_i, a_k)$. Further, we define $z \coloneqq \max_{c_j \in C} x_j^c$; that is, *z* is the maximum log likelihood that any citizen does not respond to the vaccine (or, alternatively, the minimum log likelihood that any citizen will respond to the vaccine). Finally, then, our aim is to minimize *z*.

ILP formulation. Our ILP formulation consists of three types of variables:

- x_i^v: one binary indicator variable for each vaccine element which indicates whether it is included in the vaccine for the given population. We usually index vaccine elements with *i*.
- x_j^c: one continuous variable for each citizen in the population which gives the log likelihood of no response for that citizen. We always index citizens with *j*.
- x_k^a: one continuous variable for each HLA allele which gives the log likelihood of no response for that allele. We always index alleles with *k*.
- z: one continuous variable which gives the maximum log likelihood that any citizen does not respond to the vaccine. (Our goal will be to minimize this value.)

Additionally, the ILP uses the following constants:

- $p_{i,k}$: the log likelihood that vaccine element v_i does not cause a response for allele k.
- c_i^v: the “cost” of vaccine element v_i.
- b: the maximum cost of vaccine elements which can be selected.

Finally, the ILP uses the following constraints:

- $x_k^a = \sum_i p_{i,k} \cdot x_i^v$: one constraint for each allele which gives the log likelihood that at least one selected peptide results in a positive response for that allele
- $x_j^c = \sum_{a_k \in A(c_j)} x_k^a$: one constraint for each citizen which gives the log likelihood that at least one selected peptide results in a positive response for at least one allele for that citizen. (That is, this is the likelihood of a positive response for this citizen.)
- $b \geq \sum_i c_i^v \cdot x_i^v$: the vaccine elements we select cannot exceed the budget
- $z \geq x_j^c$: as discussed above, we use *z* as an approach to solve the minimax problem. These constraints imply that *z* is the minimum log likelihood that any individual patient will respond to the vaccine.

Objective: The objective of the ILP is to minimize *z*.

The setting of the binary x_i^v variables corresponds to the optimal choice of vaccine elements for the given population.

Relationships to max-flow and other problems with provably efficient solutions. This is highly-related to a number of efficiently solvable network flow problems. Our problem is essentially a min-flow problem with multiple sinks, where each citizen is a sink; however, our aim is to minimize the flow to each individual sink rather than the flow to all sinks. In particular, rather than the “sum” operator typically used to transform multiple sink flow problems into a single-sink problem, we would need a (non-linear) “min” operator. Thus, efficient min-flow formulations are not applicable in this setting.
